# Improving the quality of data in clinical trials in cancer. COMPACT Steering Committee.

**DOI:** 10.1038/bjc.1991.95

**Published:** 1991-03

**Authors:** 


Br. J. Cancer (1991), 63, 412-415 © Macmillan Press Ltd., 1991

SHORT COMMUNICATION

### Improving the quality of data in clinical trials in cancer

COMPACT Steering Committee*

Clinical trials are widely used as a means to test the efficacy of treatments in cancer. The main statistical requirements of a successful Phase III clinical trial are described by Peto *et al.* (1976) as:

(i) that patients are allocated their treatment in such a way as to avoid bias in the treatment comparison – this requires some form of randomisation;
(ii) that the number of patients entered is sufficiently large to give a reasonable chance of detecting a clinically worthwhile difference; and
(iii) that the data gathered from the trial have a high level of reliability and completeness.

The first two of these requirements have received lengthy discussion in the literature (Pocock, 1983; Buyse *et al.*, 1984; Freedman, 1989). Thus an international survey of cancer trials conducted on behalf of the Worth Health Organisation (WHO) has shown that, while randomisation is now commonly employed, it is less usual to find adequate numbers of patients in trials (Pocock, 1978). This was confirmed to be the case in the United Kingdom (Tate *et al.*, 1979). Realisation of this lack of patient numbers has led to greater efforts to increase trial size, with the formation of national and international cooperative groups such as the United Kingdom Childhood Cancer Study Group (UKCCSG), United Kingdom Coordinating Committee for Cancer Research (UKCCCR) and the European Organisation for Research and Treatment of Cancer (EORTC), to organise multicentre studies. Commensurate with this effort, there has been a growth in the number and size of Cancer Trials Offices, providing statistical, computing and data management support, to coordinate the conduct of these trials.

Less attention has been paid to the third requirement of data reliability and completeness. Along with the growth in size of clinical trials comes a corresponding increase in the amount of data handling although an established principle, particularly in large studies, is to collect only information germane to the main question under investigation which is often survival.

Nevertheless, in cancer trials follow-up is often prolonged with the consequential accumulation of substantial data per patient. In addition survival time may not constitute the only endpoint of interest. For example, in adjuvant studies where there may be only a small survival benefit for a drug, the longitudinal assessment of toxicity from chemotherapy is particularly important. Therefore, as well as initial details and disease characteristics of each patient, and the treatment given, cancer trials often require the recording of substantial follow-up information including toxicity data and measures of quality of life. The volume of data collected makes quality control a real problem and one that needs careful planning from the outset. These considerations should influence the clarity of the trial protocol, the corresponding data forms and instructions to personnel involved in data collection and recording.

It is clear that conclusions drawn from a clinical trial depend on the quality of the patient data collected, so it is important that the data are accurate and complete. This is often one aspect of any study for which external validation by, for example, a journal referee, is not possible unless the individual patient data are provided in a suitable format. One consequence is that questions on data accuracy and completeness are omitted from published checklists of the statistical content of medical studies (Gardner *et al.*, 1986). In contrast such checklists ask direct questions about randomisation and the study numbers necessary to provide sufficient statistical power. Answers to these latter questions should be clear and unambiguous from the text and can be verified, at least indirectly, by a reviewer.

A central theme in the development of a successful strategy to maintain data quality is the designation of a person (or team) responsible for the overall co-ordination of data collection, an activity that has become entitled 'data management'. Since effective analysis of cancer trials requires access to a computer based statistical analysis package, data management in a trial extends to maintenance of an accurate computerised database. To achieve this, automated data quality procedures to aid the detection, review and correction of erroneous values are essential (Friedman *et al.*, 1983; Karison, 1981; Kronmal *et al.*, 1978). The advent of modern technology may now permit direct entry into the trial data bank. Randomisation is already available through EuroCODE (1990) to, for example, EORTC and some Cancer Research Campaign (CRC) and Medical Research Council (MRC) cancer trails. This facility merely re-emphasises the problem of data quality and raises problems of security and confidentiality.

Data from a multicentre clinical trial should be collected on forms specifically designed for the trial. Typically each patient entered has an entry form, a variable number of follow-up forms and possibly some 'special' single forms. As an example, consider the International Collaborative Cancer Group (ICCG) trial of adjuvant 5- Fluorouracil, Adriamycin and Mitomycin C in operable gastric cancer (Coombes *et al.*, 1990). For this trial the initial clinical data form records demographic details and medical history of the patient, while two 'special' forms record histology and staging of the tumour and date of randomisation and the particular treatment doses allocated. Two additional 'special' forms record dates and details of first distant metastases and death respectively. Thus there is an initial form, four special forms and repeated follow-up forms. The forms are printed in booklets on NCR paper and allow the top copy to be sent to the Trials Office, where they will be processed to form a computerised data base for the study in question. A duplicate copy of the form is kept in the patients' notes.

An important determinant of success in a multicentre trial

*Steering Committee members: J.M. Bliss, E. Heath, Section of Epidemiology, Institute of Cancer Research, Block D, 15 Cotswold Road, Belmont, Sutton, Surrey SM2 5NG; T.N. Bryant, Medical Statistics and Computing, University of Southampton, Southampton General Hospital, Southampton SO9 4XY; C.E.D. Chilvers, Department of Public Health Medicine and Epidemiology, University of Nottingham Medical School, Queen's Medical Centre, Nottingham NG7 2UH; P.M. Fayers, D. Machin, Medical Research Council Cancer Trials Office, 7 Green Street, Cambridge CB2 3JU; R.M. Greenwood, Department of Computing, Royal Marsden Hospital, Fulham Road, London SW6 3JJ and A.J. Westlake, Small Area Health Statistics Unit, London School of Hygiene and Tropical Medicine, Keppel Street, London WC1E 7HT, UK.
Correspondence: J.M. Bliss.
Received and accepted 10 September 1990.

is the ability to give participating investigators regular information on the progress of a trial so as to maintain their interest. This usually occurs at regular meetings of participants at which interim reports are presented. These reports should include the most recently collected data that have nevertheless undergone checks for errors and inconsistencies. This is particularly important if the trial has an associated data monitoring committee empowered to cease recruitment to a trial prematurely if circumstances warrant it.

The ICCG trial requires immediate feedback of untoward toxicity to the clinicians. Other groups may wish the study coordinators alone to be informed immediately of treatment related deaths, perhaps particularly those associated with an event recorded on an earlier visit form for that patient. Both of these requirements demand rapid data entry so that all information received at the Trials Office must be immediately entered on the data base. In this way the latest information on a particular patient is linked to the earlier data available on that patient.

In anticipation of the receipt of the first complete patient form from the clinical trial an appropriate data base has to be prepared. This is now done for many trials conducted on behalf of the MRC, CRC, UKCCSG and UKCCCR, using COMPACT (COMputer PAckage for Cancer Trials) a menu driven data management package designed on behalf of the MRC and CRC specifically for improving the quality of data in cancer clinical trials that involve detailed patient follow-up.

An important feature of COMPACT is the use of a file recording the problems arising from forms. All data, whether correct, queried or incorrect, are entered on the computer but those data that are queried, or are known to be incorrect during the entry process, are automatically marked as being suspect and details are recorded in the reserved problems file. This avoids the accumulation of data forms which may contain important information in the Trials Office, awaiting replies concerning less critical items from clinical investigators.

The sequence of events in setting up a trial in COMPACT begins with a definition of the types of forms used and the content of each form. This process is performed interactively, and at the same time error checks can be defined. These error checks are primarily of two types. Thus checks are used to detect values outside defined ranges, for example, normal haematological ranges or to verify that patients indeed satisfy the protocol requirements for entry to the trial. In addition checks are able to test for inconsistencies between separate data items both within and across data forms from a particular patient.

A particularly powerful use of the consistency checking procedure is the ability to compare values at successive visits. The facility to make such cross checks is a strong tool for quality control. For example, although a white blood cell count of $4.0 \times 10^9 l^{-1}$ recorded at a particular visit would be unlikely to violate a range check, if the same patient had had a count of $8.0 \times 10^9 l^{-1}$ a month previously this would either cast doubt on one of the readings or signal a rapid deterioration in the patient. Thus although both 4.0 and $8.0 \times 10^9 l^{-1}$ would pass a range check, the combination would signal a problem, which may or may not be an error, for that patient. Consistency checks can be as complicated as the protocol demands.

Examples of error checks are given in Table I for an adjuvant breast cancer study which requires the patients to be aged between 18 and 70 and have an initial white blood cell count (WBC) of at least $4.0 \times 10^9 l^{-1}$. There is also a requirement that any patient experiencing weight loss of more than 10% during treatment should be identified.

Thus the first part of Table I calculates the age of women at randomisation and then checks if she is between 18 and 70 years of age. If she is not then a message is displayed. There is also a check on the range for the initial WBC value. The consistency check for weight loss, first compares the weight at the first follow-up visit with the initial weight and calculates the percentage change. For subsequent follow-up visits (referred to as THIS visit) a comparision is made with the previous visit (THIS-1). THIS therefore corresponds to the actual and latest, follow-up information being entered by the Data Manager. At the follow-up visit on 10th January 1990 patient number 1005 had recorded a 20% weight loss since the previous follow-up visit.

Thus such checks can be used to identify patients that the protocol specifies require the Trial Co-ordinator's, rather than just the Data Managers, immediate attention. For example, those who receive a modified dosage schedule, or experience a particular form of unexpected toxicity or as in the above example a serious weight loss. Once the forms and checks are defined, interactive data entry can begin. In COMPACT this is carried out by selection from a menu of items until the relevant form for the particular patient is identified. The items on the form are then displayed one at a time and the data directly input. The data input program is designed for ease of use by those without computer programming expertise. Should a data item entered violate an error check, the system generates a REJECT message. This immediately alerts the Data Manager to check the entry made against the paper form. Should the item actually recorded on the form provoke a major range or consistency violation or if the data item is missing, this is recorded in the separate PROBLEMS file, and the patient data file is flagged at an appropriate point.

At the end of the data entry session the PROBLEMS file can be inspected and appropriate action taken. It is usually the case in practice that many of these problems can be easily resolved with the participating centre, perhaps by a telephone call, and the Data Manager will enter the corrected values at the next data entry session. If these new values do not violate range or consistency checks, the system automatically deletes the relevant entry in the PROBLEMS file. In some circumstances a flag can be entered in the PROBLEMS file indicating in effect, 'This is an apparently abnormal value, that lies outside our range checks but we have verified with the investigator who confirms that it is abnormal but correct.' Once flagged in this way future error messages about this value will be automatically suppressed and the value passed to the main data file. Such a device leaves an appropriate explanation of the apparent anomaly on the data file for future reference. At any stage such aberrant values can be displayed from the PROBLEMS file if appropriate.

The PROBLEMS file is a unique feature of COMPACT which is not available in other data management software since most have not been designed specifically for clinical trial use.

Another important aspect of trial management is checking that patients are seen regularly and that their progress reports are returned on schedule. COMPACT provides the facility to check the most complex of follow-up schedules, and signal overdue information. Thus the later part of Table I shows that the visit on 10 March 1990 for patient number 1005 was not at the schedule anticipated of 6 weeks following her previous visit of 10 January 1990.

COMPACT has been designed to act an an interface between the clinical trial data and standard statistical analysis packages. However, most statistical packages can only process rectangular (flat) data files while the data collected in a clinical trial are always 'ragged', because patients enter a trial at different times and have different numbers of follow-up visits depending on the length of their survival. When COMPACT generates an output data file for analysis it also automatically produces the appropriate data description and format required for the particular statistical package and the specified variables of interest for analysis. This facility substantially reduces the amount of programming involved in producing an analysis. It can also select particular subgroups of patients or follow-up examinations and derive new variables, such as patient survival time from randomisation both for patients who have died and for those still alive (censored). If repeat analyses are to be carried out for successive collaborators meetings, perhaps to report on the toxicity experience of the patients, then an updated flat data file and output specifications can be regenerated using the stored instructions from the first analysis.

An example of commands for the calculation of survival time and the creation of an analysis file for ultimate transfer to a statistical package is given in Table II. Thus if patients are still alive the latest date known to be alive will either be on the last follow-up form (referred to as LEXAM) or on the relapse form. For those patients who have died the date of death is recorded on a separate 'death' form.

There are many facets that govern the quality of data submitted to a trials office: these include the clarity of the data forms, the level of enthusiasm of the participants and the nature of the information requested. While we have not discussed these aspects in detail they are important and must not be overlooked. Mistakes and omissions in the data are bound to occur, however well designed the trial and however conscientious the investigators.

Some organisations, for example the EORTC and some of the Cooperative Groups in the USA, have established data quality committees. These are concerned not only about the quality of the treatment actually given, for example how closely the investigators adhere to the study protocol, but how the contents of the patient records and study data forms compare. Such committees are not discussed in detail here. However important their role, it is clear that they can only sample 'quality' from time to time. In contrast, most would agree that rapid data entry, with associated immediate signalling of queries on a daily basis, is a vital component of overall data quality.

It is our view that data entry to a clinical trial is best done, patient form by patient form, by a Data Manager who is conversant with the trial protocol and who is also responsible for reporting on the progress of the trial to the investigators. Thus the data will be entered by someone who knows the study intimately, the individual patients, albeit indirectly, and the data checking facilities themselves will have been designed with their assistance and experience from other trials. This is perhaps one of the most important guarantees of data quality.

**Table I** Error checks for an adjuvant breast cancer trial

| COMPACT code* | COMPACT message if check is violated |
|---|---|
| *Checks – range* | |
| *AGE* = TRUNC ((**DOR** – **DOB**)/365.25)<br>IF *AGE* NOTIN (18-70)<br>REJECT 'Patient aged = ',*AGE*,' – protocol violation' | 'Patient aged = 72 – protocol violation' |
| IF **WBCINIT** LT 4.0<br>REJECT 'Initial WBC = ',**WBCINIT**,' – protocol violation' | 'Initial WBC = 3.2 – protocol violation' |
| *Checks – consistency* | |
| *CHWT* = **WTINIT** – **WTFU**(1)<br>*CW* = **WTFU**(THIS-1) – **WTFU**(THIS)<br>*PERC* = (*CHWT*/**WTINIT**)*100<br>*PCTW* = (*CW*/**WTFU**(THIS-1))*100 | |
| IF(ABS(*PERC*) GE 10.5) AND (**FU**(1) EQ '1')<br>REJECT 'Weight change > 10% Initial = ',WTINIT, 'kg. Present = ',**WTFU**(1),'kg.'<br>IF ABS(*PCWT*) GE 10.5<br>REJECT 'Weight change > 10% Previous = ',**WTFU**(THIS-1),'kg, Present = ',**WTFU**(THIS),'kg Change = ', *PWCT*,'%' | ID 1005, Visit date 10.01.90: Weight change > 10%, Previous = 70 kg, Present = 56 kg Change = 20% |
| *Checks – schedule* | |
| *DELAY* = **VISIT**(THIS)–**VISIT**(THIS-1)<br>IF (*DELAY* > 42) AND (**TREAT** EQ '1')<br>REJECT *DELAY*,' days between visits for patient receiving Treatment 1, too long' | ID 1005, Visit date 10.03.90: 59 days between visits for patients receiving Treatment 1, too long |

*COMPACT variable names in **BOLD**, derived variable names in *ITALICS*.

**DOR** = Date of randomisation.

**DOB** = Date of birth.

**VISIT** = Date of visit.

**FU** = Visit number.

**WBCINIT** = Initial WCB count.

**WTINIT** = Initial weight.

**WTFU** = Weight at follow-up examination

**ID** = Patient identifier.

**Table II** Commands to calculate survival time for an adjuvant breast cancer study

| *COMPACT code** | *Comment* |
|---|---|
| *DLS* = MAX (**DREL**,**DFU**(LEXAM)) | Calculate latest date that patients were seen alive (whether at most recent routine follow-up or at relapse) |
| IF **DOD** NE **MISS**<br>BEGIN | Calculating survival times and calculating status variable |
| *SURV* = **DOD** – **DOR**<br>*STATUS* = 1 | – For patients who have died (*STATUS* = 1), survival time is from randomisation to death |
| ELSE<br>*SURV* = *DLS* – **DOR**<br>*STATUS* = 0<br>ENDIF | – For patients who are alive (*STATUS* = 0), survival time is from randomisation to the date they were last known to be alive and is censored |
| FILE **ID**, **TREAT**, *SURV*, *STATUS* | Output derived data with relevant variables to a file for statistical analysis |

*COMPACT variables names in **BOLD**, derived names in *ITALICS*

**DOR** = Date of randomisation

**DFU** = Date of follow-up (repeated)

**DREL** = Date of relapse

**DOD** = Date of death

**ID** = Patient identifier

**TREAT** = Treatment regimen

In some situations batch entry is unavoidable. For example, in very large trials with large numbers of patient forms arriving daily the data are often entered into the computer file by a 'Keypunch Operator' rather than a 'Data Manager'. In these circumstances patient form by patient form checking procedures at data entry may be entirely absent or at least will be less efficient, perhaps resulting in a proportionally larger number of queries being generated. However such data can be entered into COMPACT in batch mode, and the resulting queries addressed by means of the PROBLEMS file.

The increasing awareness of the need for larger collaborative trials for certain tumour types has resulted in cooperative group trials, and they may be organised in such a way that there is more than one Trials Office involved in the randomisation and data management processes. Such collaboration demands a uniform pattern of data checking and data files that are transferable between sites.

For large studies automated quality control is essential and many Trials Offices have developed their 'in-house' methods for this purpose. The computer software for these systems, however, has generally not been transferable to other working environments. This contrasts sharply with the widespread use of standard statistical analysis packages which have been available on a wide range of computers for many years. There are considerable advantages in using a tried and tested data management package rather than writing software for every new trial, which is an expensive and time consuming operation.

Most data management packages that can be purchased have not been designed specifically for clinical trial use. They tend either to be large, expensive and available only on large computers, or rather basic, resulting in the need for considerable extra development. Most are also difficult for inexperienced computer users. Moreover, we are not aware of any with the vital PROBLEMS and longitudinal data facilities or adequate means of dealing with missing data.

COMPACT has been written with cancer trials in mind and certain fundamental aspects of good clinical trial practice are embedded in the package. Thus there are facilities to monitor the incoming data and provide rapid feedback to the trial co-ordinator, enabling corrective action to be taken when problems arise. One can equally well use COMPACT for Phase II trials in cancer, for trials of treatment for other diseases, or for other types of clinical or laboratory studies requiring follow-up. COMPACT has also been successfully used for epidemiological studies and for teaching purposes in courses involving clinicans and medical statisticians.

There are no constraints on the number of patients that can be entered into a study using COMPACT, apart from overall disc capacity on the computer being used. On a micro computer running MS-DOS, where memory is limited, up to eight forms, 300 variables and 20 visits are possible. There is a detailed user manual. COMPACT is currently in use at ten centres in the United Kingdom running in excess of 100 clinical trials, more than half of which are in cancer. A second generation version of COMPACT is under development.

We believe that COMPACT is particularly suitable for use in smaller Trials Offices where adequate computer programmer support cannot be provided to develop an 'in-house' system. COMPACT can be up and running for a new trial in a matter of days, the exact time depending on the experience of the programmer or data manager and the complexity of the trials forms and follow-up schedules. Organisers of new studies should consider COMPACT before they go to the expense of developing their own computer software. It is available at low cost for CRC and MRC supported groups. Information about availability and support for users may be obtained from J.M. Bliss.

COMPACT has received support from the Cancer Research Campaign and the Medical Research Council.

### References

BUYSE, M.E., STAQUET, M.J. & SYLVESTER, R.J. (1984). *Cancer Trials: Method and Practice*, Oxford University Press: Oxford.

COOMBES, R.C., SCHEIN, P.S., CHILVERS, C.E.D. & 17 others on behalf of the International Collaborative Cancer Group (1990). A randomised trial comparing adjuvant 5-fluorouracil, adriamycin and mitomycin C (FAM) with no treatment in operable gastric cancer. *J. Clin. Oncol.*, **8**, 1362.

EUROCODE STEERING COMMITTEE (1990). EuroCODE: a new approach to collaborative research in clinical oncology. *European J. Cancer & Clin. Oncol.*, **25**, 1905.

FREEDMAN, L.S. (1989). The size of clinical trials in cancer research – what are the current needs? *Br. J. Cancer*, **59**, 396.

FRIEDMAN, R.B., ENTINE, S.M. & CARBONE, P.P. (1983). Experience with an automated cancer protocol surveillance system. *Am. J. Clin. Oncol.*, **6**, 583.

GARDNER, M.J., MACHIN, D. & CAMPBELL, M.J. (1986). Use of checklists for the assessment of the statistical content of medical studies. *Br. Med. J.*, **292**, 810.

KARISON, T. (1981). Data editing in a clinical trial. *Cont. Clin. Trials*, **2**, 15.

KRONMAL, R.A., DAVIS, K., FISHER, L.D., JONES, R.A. & GILLESPIE, M.J. (1978). Data management for a large collaborative clinical trial (Cass: Coronary Artery Surgery Study). *Comp. Bio. Res.*, **11**, 553.

PETO, R., PIKE, M.C., ARMITAGE, P. & 7 others (1976). Design and analysis of randomised clinical trials requiring prolonged observation of each patient: Introduction and design. *Br. J. Cancer*, **34**, 585.

POCOCK, S.J. (1978). The size of cancer trials and stopping rules. *Br. J. Cancer*, **38**, 757.

POCOCK, S.J. (1983). *Clinical Trials: A Practical Approach*. Wiley: Chichester.

STAQUET, M., SYLVESTER, R., MACHIN, D. & 8 others (1977). The EORTC Data Center. *Eur. J. Cancer*, **13**, 1455.

TATE, H.C., RAWLINSON, J.B. & FREEDMAN, L.S. (1979). Randomized comparative studies in the treatment of cancer in the United Kingdom: room for improvement. *Lancet*, **ii**, 623.


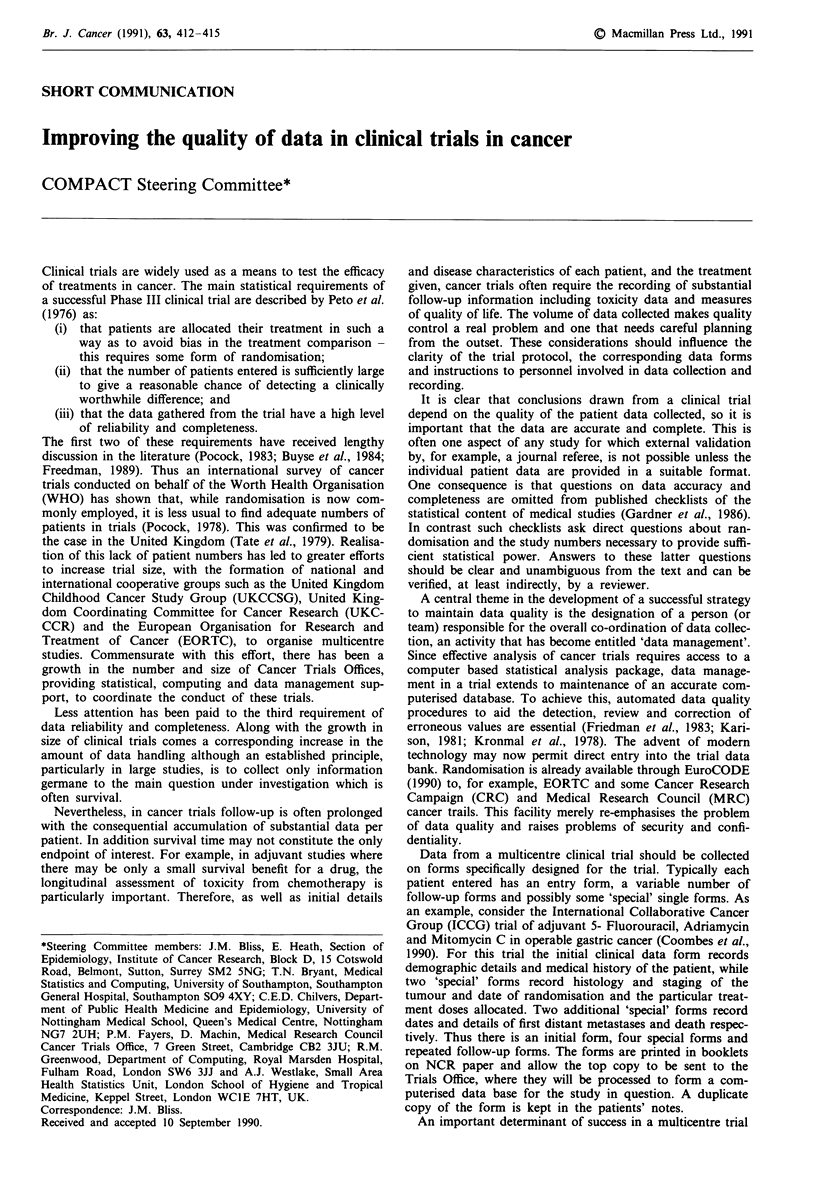

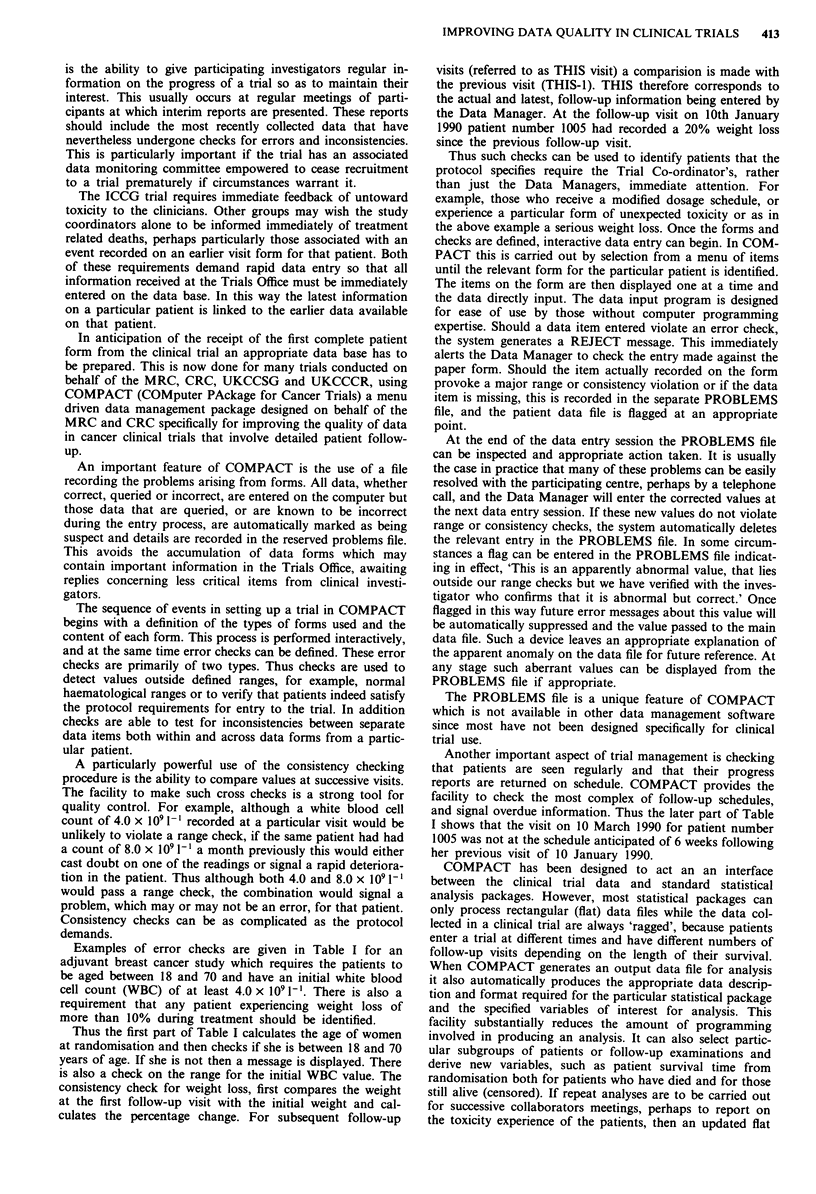

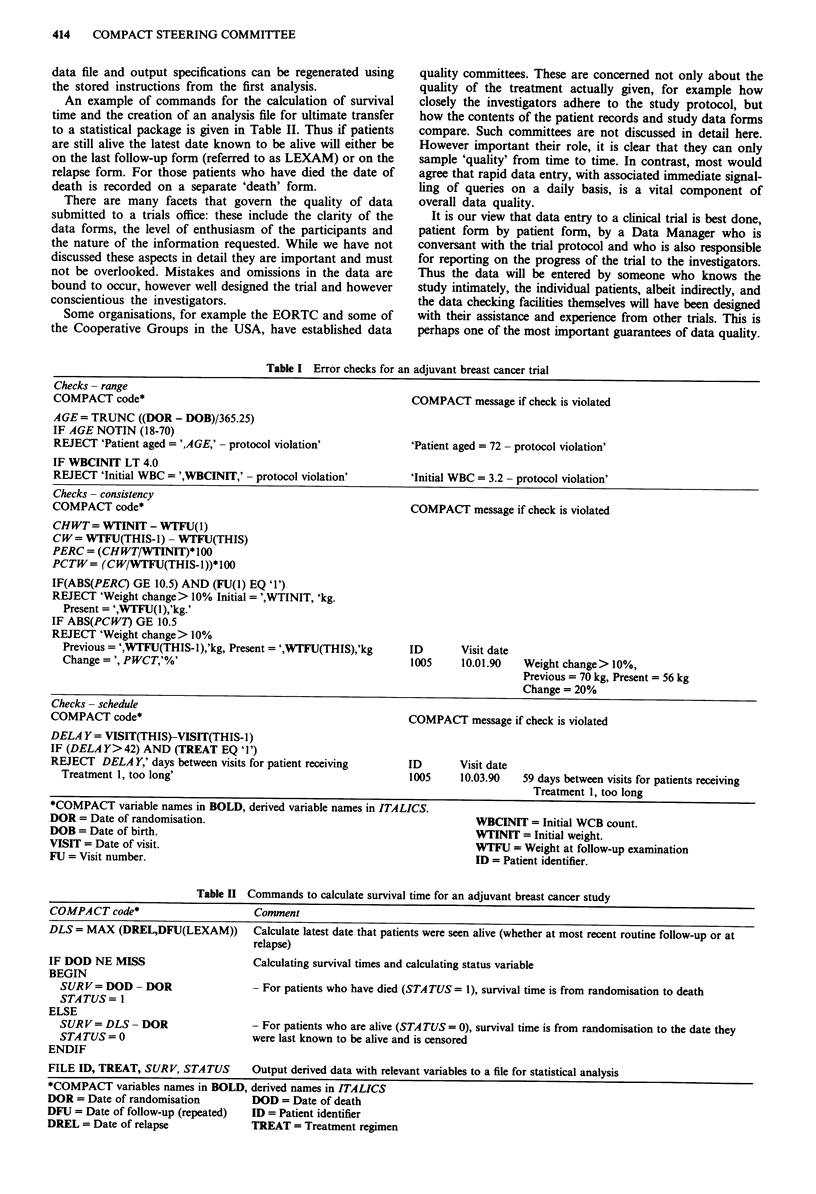

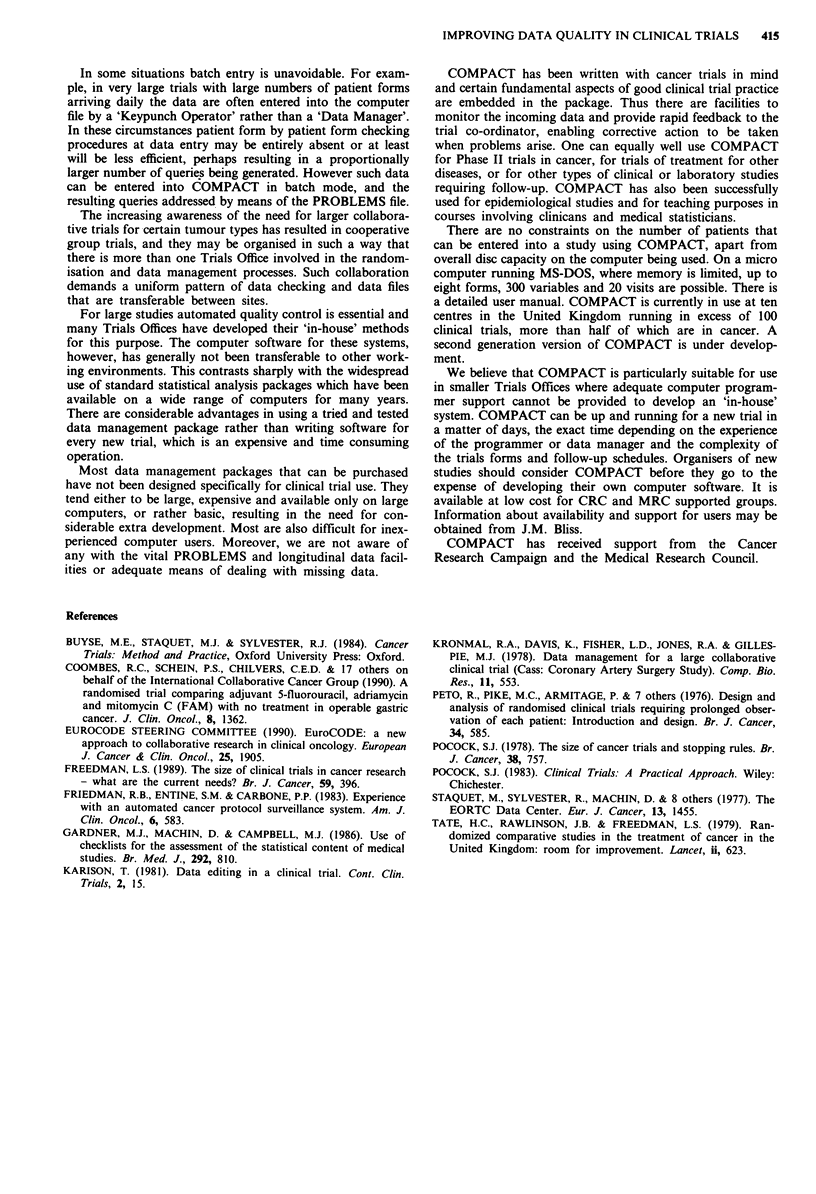


## References

[OCR_00541] Coombes R. C., Schein P. S., Chilvers C. E., Wils J., Beretta G., Bliss J. M., Rutten A., Amadori D., Cortes-Funes H., Villar-Grimalt A. (1990). A randomized trial comparing adjuvant fluorouracil, doxorubicin, and mitomycin with no treatment in operable gastric cancer. International Collaborative Cancer Group.. J Clin Oncol.

[OCR_00553] Freedman L. S. (1989). The size of clinical trials in cancer research--what are the current needs? Medical Research Council Cancer Therapy Committee.. Br J Cancer.

[OCR_00557] Friedman R. B., Entine S. M., Carbone P. P. (1983). Experience with an automated cancer protocol surveillance system.. Am J Clin Oncol.

[OCR_00562] Gardner M. J., Machin D., Campbell M. J. (1986). Use of check lists in assessing the statistical content of medical studies.. Br Med J (Clin Res Ed).

[OCR_00577] Peto R., Pike M. C., Armitage P., Breslow N. E., Cox D. R., Howard S. V., Mantel N., McPherson K., Peto J., Smith P. G. (1976). Design and analysis of randomized clinical trials requiring prolonged observation of each patient. I. Introduction and design.. Br J Cancer.

[OCR_00583] Pocock S. J. (1978). Size of cancer clinical trials and stopping rules.. Br J Cancer.

[OCR_00591] Staquet M., Sylvester R., Machin D., van Glabbeke M., de Grauwe G., Wennerholm A., Tyrrell J., Renard J., de Pauw M., Eeckhoudt D. (1977). The E.O.R.T.C. Data Center.. Eur J Cancer.

[OCR_00595] Tate H. C., Rawlinson J. B., Freedman L. S. (1979). Randomised comparative studies in the treatment of cancer in the United Kingdom: room for improvement?. Lancet.

